# Beyond Antioxidant Scavenging: Mediterranean Diet, Redox–Metabolic Integration, and Exercise Adaptation

**DOI:** 10.3390/biotech15040088

**Published:** 2026-10-06

**Authors:** Stefania D’Angelo

**Affiliations:** Department of Medical, Human Movement, and Well-Being Sciences (DiSMMeB), University of Naples Parthenope, 80133 Naples, Italy; stefania.dangelo@uniparthenope.it

**Keywords:** Mediterranean diet, dietary bioactives, polyphenols, bioavailability, exercise adaptation, redox signaling, gut microbiota, nutraceuticals

## Abstract

Exercise-induced reactive oxygen and nitrogen species (RONS) are not merely damaging by-products but essential components of signaling networks that support training adaptation. This narrative review examines how the Mediterranean diet (MedDiet) may modulate exercise-responsive redox and metabolic signaling beyond direct antioxidant scavenging. Evidence from mechanistic, preclinical, observational, and controlled human studies was evaluated, with attention to dietary patterns, whole foods, standardized extracts, and isolated supplements. Biological effects depend on food composition, matrix, dose, timing, intestinal absorption, host metabolism, microbial biotransformation, and the resulting circulating metabolite profile. Polyphenol conjugates, microbiota-derived phenolic metabolites, short-chain fatty acids, and lipid-derived mediators may interact with redox, energy-dependent, inflammatory, and vascular pathways engaged by exercise. Human evidence remains heterogeneous and suggests possible benefits for selected endurance-related, metabolic, vascular, and recovery-related outcomes, although evidence for accelerated recovery remains limited, rather than supporting a consistent ergogenic effect. Microbiota-derived metabolites are plausible mediators, although causal evidence remains limited. High-dose antioxidant supplementation may attenuate selected redox-sensitive responses and should not be considered equivalent to antioxidant-rich foods. Overall, an exposure-aware, food-first framework may better explain diet–exercise interactions. Future studies should integrate dietary characterization, circulating metabolite assessment, standardized training loads, and temporally resolved molecular and functional outcomes to advance precision redox nutrition in physically active populations and athletes across training contexts.

## 1. Introduction

Exercise training elicits a coordinated network of redox, energy-dependent, calcium-dependent, and mechanical signals that drive tissue remodeling and functional adaptation. In contracting skeletal muscle, transient increases in reactive oxygen and nitrogen species (RONS) contribute to the regulation of mitochondrial biogenesis, endogenous antioxidant defenses, substrate utilization, and vascular responses. Exercise-derived RONS should therefore not be regarded merely as damaging by-products: their biological effects depend on concentration, subcellular localization, and duration, and moderate, spatially controlled redox fluctuations are integral to adaptive signaling [1]. Accordingly, redox homeostasis during exercise reflects a dynamic balance between physiological RONS-dependent signaling and the prevention of excessive or unresolved oxidative stress.

This biphasic role of RONS creates a fundamental challenge for nutritional research. Excessive or unresolved oxidative stress can impair cellular function and recovery, whereas indiscriminate suppression of exercise-induced redox signals may attenuate pathways required for training adaptation. The relevant question is therefore not whether dietary bioactives with antioxidant or redox-modulating properties suppress RONS, but whether they modulate the magnitude, timing, and resolution of redox responses while preserving their physiological signaling functions. This distinction is particularly important when antioxidant-rich dietary patterns are compared with isolated compounds administered at pharmacological doses [2]. From this perspective, the potential nutritional benefit lies in modulating the redox environment rather than indiscriminately eliminating exercise-induced reactive species.

The Mediterranean diet (MedDiet) provides a valuable model for investigating this question. Its characteristic combination of vegetables, fruits, legumes, whole grains, nuts, extra-virgin olive oil, and fish supplies polyphenols, unsaturated fatty acids, dietary fiber, vitamins, minerals, and other bioactive constituents within complex food matrices. These components may interact with exercise-responsive pathways involving Nrf2–Keap1 signaling, NF-κB-dependent inflammatory regulation, AMPK–SIRT1–PGC-1α-mediated mitochondrial remodeling, endothelial nitric oxide signaling, and microbial metabolism [3,4,5,6,7]. The biological activity of the MedDiet therefore extends beyond the direct chemical scavenging of free radicals [2].

Human intervention studies suggest that adherence to the MedDiet may favor selected outcomes related to endurance performance, muscle strength, body composition, and antioxidant status. However, the evidence remains limited and heterogeneous because studies differ substantially in dietary protocols, study populations, exercise modalities, intervention duration, comparator diets, and outcome selection [8,9]. Moreover, mechanistic interpretations are frequently extrapolated from studies of isolated compounds or from experimental concentrations that do not reflect the bioavailability, metabolism, and tissue exposure of dietary bioactives in humans.

The gut microbiota introduces an additional source of biological complexity and interindividual variability. Fermentable dietary fiber reaching the colon can be metabolized by microbiota to short-chain fatty acids, whereas poorly absorbed polyphenols can undergo microbial biotransformation into smaller phenolic metabolites. The MedDiet may also alter microbial community structure and metabolic activity. These processes may influence systemic inflammation, energy metabolism, intestinal barrier function, and skeletal muscle physiology. Recent human evidence indicates that a MedDiet intervention during endurance training can modify gut microbial composition and increase circulating propionate and butyrate in parallel with improvements in maximal oxygen uptake. Nevertheless, the observed associations and mediation analyses do not establish that these metabolites directly cause the improvement in training adaptation [10].

Existing reviews have generally addressed individual components of the diet–exercise relationship, including antioxidant status, performance, inflammation, mitochondrial function, or the gut microbiota [2,8,9]. A critical synthesis integrating these dimensions within a unified redox–metabolic framework remains limited. In particular, it is unclear whether the MedDiet primarily reduces excessive oxidative burden or instead fine-tunes exercise-responsive signaling through the coordinated effects of food-derived bioactives, their circulating metabolites, and microbiota-dependent transformations.

### What This Review Adds Beyond Previous Reviews

Recent reviews have evaluated the relationship between the Mediterranean diet and athletic performance, strength, body composition, antioxidant status, and sport-related health outcomes, while other syntheses have addressed gut microbiota–exercise interactions or the bioavailability and biological activity of dietary polyphenols [8,9,11,12]. However, these dimensions have generally been considered separately or primarily from the perspective of dietary adherence and functional outcomes. A comparison of the scope, focus, and conceptual contribution of the present review with selected recent reviews published between 2024 and 2026 is provided in Supplementary Table S1.

The distinctive contribution of the present review is therefore integrative rather than based on the proposal of a new isolated molecular mechanism. It introduces an exposure-aware framework linking the composition and form of the ingested dietary exposure with food-matrix effects, intestinal absorption, host and microbial biotransformation, circulating metabolite profiles, exercise-responsive redox and metabolic signaling, and potential functional outcomes.

A further distinguishing feature is the explicit separation of whole dietary patterns and foods from standardized extracts and isolated high-dose supplements. These exposures may contain nominally similar bioactive compounds but differ substantially in dose, matrix, pharmacokinetics, metabolite profiles, and biological context. By integrating these factors, the review aims to distinguish mechanistic plausibility from demonstrated human efficacy and to provide a physiologically grounded framework for interpreting Mediterranean diet–exercise interactions.

Accordingly, this narrative review critically examines how MedDiet may modulate exercise-induced molecular and functional adaptations beyond direct antioxidant scavenging. Rather than addressing the broader contribution of diet–exercise interactions to healthy aging, the review focuses on exercise-specific nutritional exposures and their implications for training adaptation, performance, and recovery. Attention is given to the differences among whole dietary patterns, food matrices, isolated bioactives, and high-dose antioxidant supplements, together with the roles of bioavailability, microbial biotransformation, and interindividual variability. Evidence from cellular, animal, observational, and controlled human studies is evaluated separately to distinguish mechanistic plausibility from demonstrated efficacy. By linking ingested exposure to circulating metabolites, redox-sensitive signaling, and potential functional outcomes, the review proposes an exposure-aware framework for precision redox nutrition in exercise.

## 2. Literature Search Strategy and Evidence Appraisal Framework

This structured narrative review was designed to provide a critical and integrative synthesis of mechanistic and translational evidence on how Mediterranean dietary patterns, foods, and food-derived bioactives may influence exercise adaptation through redox, metabolic, pharmacokinetic, and microbial pathways. The literature search was used to support a transparent and focused narrative synthesis rather than to conduct an exhaustive systematic or scoping review.

The literature search covered publications indexed from January 2000 through August 2026 in PubMed/MEDLINE, Scopus, and the Web of Science Core Collection. The search was updated through August 2026. Earlier seminal studies were retained when necessary to establish foundational biochemical or physiological concepts. The reference lists of relevant reviews and eligible primary studies were screened, and forward citation tracking was used to identify additional publications not retrieved through the initial database searches.

Search strategies combined controlled vocabulary, where available, with free-text terms organized into three principal blocks: (“Mediterranean diet” OR polyphenol* OR flavonoid* OR phenolic* OR “functional food*” OR nutraceutical*) AND (exercise OR training OR athlete* OR “physical activity”) AND (“oxidative stress” OR redox OR “reactive oxygen and nitrogen species” OR RONS OR Nrf2 OR AMPK OR SIRT1 OR PGC-1α OR mitochondri* OR inflammation OR endothelial). Supplementary searches incorporated microbiota, microbiome, “short-chain fatty acid*”, urolithin*, metabolite*, bioavailability, biotransformation, and “food matrix” to capture the microbial and pharmacokinetic dimensions of dietary exposure. Search terms were adapted to the syntax and indexing system of each database. Because the objective was a structured narrative synthesis rather than a formal systematic review, database-specific search strings were used to identify the relevant literature but were not prospectively designed as a fully reproducible systematic-search protocol.

Eligible publications included English-language, peer-reviewed original studies, systematic reviews, meta-analyses, and relevant narrative reviews examining Mediterranean dietary patterns, Mediterranean foods, food-derived bioactives, or antioxidant supplements in relation to exercise-responsive molecular, metabolic, performance, or recovery outcomes. Priority was given to controlled human interventions comparing dietary patterns, whole foods, extracts, or isolated compounds and reporting exercise-related functional or mechanistic outcomes. Prospective and cross-sectional human studies were retained when they provided relevant information on dietary exposure, biomarkers, performance, or recovery. Preclinical studies were included selectively when they clarified molecular targets, tissue-specific mechanisms, metabolism, bioavailability, or dose–response relationships. Analytical food-characterization studies were considered only when they informed variability in bioactive exposure arising from cultivar, processing, maturation, or food-matrix effects.

Studies were excluded when they lacked a relevant connection between dietary exposure and exercise or redox–metabolic adaptation, reported only generic chemical antioxidant capacity without informing biological exposure, were available solely as conference abstracts or non-peer-reviewed material, or duplicated data from a more complete publication. Titles, abstracts, and potentially relevant full-text articles were assessed for their relevance to the predefined conceptual scope of the review. As this was not a systematic review, study selection was not intended to generate an exhaustive study count or a PRISMA-style flow of records, and no quantitative pooling was performed.

Evidence was interpreted using a two-dimensional framework based on both study design and type of nutritional exposure. Cell-based studies were used to identify candidate pathways and direct molecular interactions, while recognizing the limited physiological relevance of simplified systems and supraphysiological concentrations. Animal studies were considered supportive of tissue-level mechanisms, metabolism, and controlled exposure–response relationships, but not as direct evidence of human efficacy. Human observational studies were treated as evidence of association and hypothesis generation, with attention to confounding and dietary measurement error. Randomized or otherwise controlled human interventions were assigned the greatest weight when evaluating efficacy, while being appraised for sample size, intervention duration, participant phenotype, background diet, energy availability, exercise protocol, bioavailability, and outcome selection.

Evidence obtained from complete dietary patterns, individual foods, standardized extracts, and isolated compounds was evaluated separately because these exposures differ in composition, dose, food-matrix interactions, and pharmacokinetic behavior. Mechanistic plausibility, human efficacy, and functional relevance were therefore distinguished throughout the review, and causal conclusions were reserved for appropriately designed human studies. This hierarchy was used as an interpretative framework rather than as a formal risk-of-bias or certainty-of-evidence grading system. Given the objective of providing a critical conceptual synthesis rather than an exhaustive quantitative assessment, the evidence was not statistically pooled and the review was not conducted or reported as a formal systematic or scoping review.

## 3. Exercise-Induced Redox Signaling as a Context for Nutritional Modulation

Exercise adaptation originates from the simultaneous perturbation of several intracellular variables rather than from a single molecular trigger. Increased ATP turnover alters AMP/ATP and ADP/ATP ratios, repeated calcium transients activate calcium-sensitive enzymes, mechanical loading engages stress-responsive kinases, and muscle contraction modifies the cellular redox environment. These signals converge on transcriptional and post-transcriptional programs controlling substrate utilization, mitochondrial remodeling, antioxidant defense, vascular function, and muscle phenotype [13]. The magnitude, localization, and temporal coordination of these initial perturbations influence the resulting molecular and functional response.

Contracting skeletal muscle generates reactive oxygen and nitrogen species through several sources, including NADPH oxidases, mitochondrial electron transport, xanthine oxidase, and nitric oxide synthases. Their relative contributions vary according to exercise modality, intensity, duration, substrate availability, and training status. RONS therefore do not constitute a uniform molecular pool: their reactivity, subcellular localization, and lifetime determine the biological targets they can modify. Transient and compartmentalized increases can participate in physiological signaling, whereas sustained or poorly controlled production can promote lipid, protein, and nucleic acid oxidation and contribute to fatigue or tissue dysfunction [1]. The distinction between adaptive redox signaling and oxidative damage is consequently quantitative, spatial, and temporal rather than absolute.

At physiologically relevant levels, RONS can reversibly modify redox-sensitive cysteine residues in phosphatases, kinases, transcription factors, and metabolic enzymes. These modifications interact with energy-dependent, calcium-dependent, and mechanical signals to coordinate acute exercise responses with longer-term transcriptional adaptation. Redox signaling also intersects with inflammatory pathways: transient NF-κB activation contributes to normal tissue remodeling, whereas excessive or prolonged activation may delay recovery and favor a maladaptive inflammatory response. Neither oxidation nor inflammation should therefore be regarded as intrinsically detrimental; their functional significance depends on magnitude, duration, and effective resolution [1,13].

The Keap1–Nrf2 system represents an important interface between exercise-induced redox perturbation and endogenous cellular defense. Under basal conditions, Keap1 promotes Nrf2 ubiquitination and proteasomal degradation. Oxidative or electrophilic modification of reactive Keap1 cysteines reduces Nrf2 turnover, facilitating its nuclear accumulation and transcriptional activity. Nrf2 subsequently promotes the expression of genes involved in glutathione synthesis, thioredoxin-dependent defense, NADPH regeneration, detoxification, and cellular repair [3]. Repeated exercise-induced activation of this pathway can strengthen endogenous antioxidant capacity and improve the response of skeletal muscle to subsequent exercise bouts [14].

Energy stress simultaneously activates AMP-activated protein kinase (AMPK), which promotes ATP-generating processes and contributes to mitochondrial adaptation through peroxisome proliferator-activated receptor-γ coactivator 1α (PGC-1α). Changes in NAD^+^ availability can influence the deacetylase SIRT1 and thereby modulate PGC-1α activity, while calcium/calmodulin-dependent kinases and mitogen-activated protein kinases provide additional regulatory inputs. These pathways form an interconnected network rather than a linear signaling sequence. AMPK-dependent changes in mitochondrial remodeling affect subsequent RONS generation, whereas Nrf2-dependent defenses influence the redox environment in which metabolic enzymes and transcriptional regulators operate [13].

This signaling network provides the physiological context in which nutritional interventions should be interpreted. Dietary bioactives may modify the amplitude or duration of selected redox, inflammatory, metabolic, and vascular responses, but they cannot substitute for the energy-dependent and mechanical signals generated by exercise. Their effects are likely to depend on chemical form, dose, timing, bioavailability, baseline nutritional status, energy availability, and training load. High-dose, rapidly bioavailable antioxidant supplements may attenuate selected redox-sensitive responses under some conditions, whereas antioxidant-rich foods produce more complex exposures shaped by the food matrix, digestion, metabolism, and microbial transformation [4]. Findings obtained with isolated supplements should therefore not be transferred directly to whole foods or dietary patterns.

Exercise adaptation can consequently be conceptualized as occurring within a context-dependent redox–metabolic window. The exercise stimulus must be sufficient to activate endogenous defenses and mitochondrial remodeling without producing unresolved oxidative or inflammatory injury. This window does not represent a fixed biochemical threshold, because it is influenced by training status, nutritional adequacy, recovery, and the characteristics of nutritional exposure. From this perspective, the potential contribution of the MedDiet lies not in eliminating exercise-induced RONS, but in modulating the physiological environment in which redox signaling, recovery, and functional adaptation occur [1,4].

The interaction among exercise load, nutritional exposure, and individual response characteristics is summarized in Figure 1. The figure distinguishes whole dietary patterns and foods from concentrated extracts and isolated supplements, emphasizing how exposure form, dose, timing, and bioavailability may contribute to differences in redox signaling and subsequent exercise-related adaptation.

## 4. Mediterranean Dietary Bioactives as Modulators of Exercise Adaptation

Within an exposure-centered framework, the MedDiet represents a complex nutritional pattern rather than a source of a single antioxidant compound. Extra-virgin olive oil (EVOO), fruits, vegetables, legumes, whole grains, nuts, and fish provide polyphenols, unsaturated fatty acids, dietary fiber, vitamins, minerals, and numerous minor constituents within structured food matrices. Repeated consumption of these foods generates a multidimensional exposure that may influence redox regulation, inflammatory resolution, mitochondrial metabolism, endothelial function, and microbial activity through complementary mechanisms [8,9]. The biological effects of the MedDiet should therefore be interpreted at the level of the dietary pattern and its resulting internal exposures rather than attributed to one compound or pathway.

Polyphenols are particularly relevant because their biological activity extends beyond direct radical scavenging. Polyphenol-rich foods and preparations have been investigated in physically active individuals in relation to oxidative stress, inflammation, exercise-induced muscle damage, recovery, and performance [5]. However, antioxidant capacity measured in a chemical assay cannot be equated with efficacy in vivo. Following ingestion, polyphenols are released from the food matrix, variably absorbed, extensively conjugated, and transformed by host enzymes and intestinal microorganisms. Tissues are therefore exposed predominantly to circulating phase II conjugates and microbiota-derived metabolites rather than to the native compounds commonly used in cell experiments. Bioaccessibility, dose, timing, metabolism, and interindividual variability must consequently be considered when translating experimental findings to habitual MedDiet consumption [6].

Studies of Mediterranean plant foods illustrate both the value and the limitations of compositional and antioxidant characterization. Polyphenol extracts obtained from the peel and flesh of Annurca apples retained antioxidant activity during reddening and ripening and protected Caco-2 cells from experimentally induced oxidative injury [15]. Subsequent profiling of peel, flesh, and core across ripening stages identified substantial differences in phenolic composition and antioxidant activity, particularly in the peel [16]. A recent narrative review further highlighted the context-dependent redox behavior of Annurca apple-derived polyphenols, bioactive fractions, and by-products, emphasizing the influence of fruit fraction, ripening stage, extract composition, dose, biological model, and cellular context on their biological effects [17]. These findings demonstrate that cultivar, tissue, maturation, and processing can modify the bioactive profile of a food. Nevertheless, chemical assays and cell-based experiments represent an early stage of the translational pathway: they cannot establish systemic bioavailability, skeletal muscle exposure, or exercise-related efficacy in humans.

The whole-diet context introduces interactions that are absent from studies of purified compounds. EVOO supplies phenolic compounds together with oleic acid, nuts and fish provide mono- and polyunsaturated fatty acids, and plant foods deliver fermentable substrates to the gut microbiota. Lipids may affect membrane composition, inflammatory signaling, and the intestinal handling of relatively hydrophobic constituents, whereas fiber and other matrix components can modify phenolic release and redirect a larger fraction toward microbial metabolism. The MedDiet should therefore be considered a coordinated exposure pattern in which polyphenols, unsaturated lipids, fiber, micronutrients, and microbial substrates may produce convergent rather than simply additive effects [6,7].

At the molecular level, MedDiet-derived compounds may interact with pathways that also respond to exercise, including Nrf2–Keap1, NF-κB, AMPK, SIRT1, and PGC-1α [3,5]. Such convergence provides biological plausibility but does not demonstrate that dietary exposure reproduces the effects observed with native compounds in experimental systems. The concentrations achieved in human tissues are generally lower, the circulating chemical forms are different, and several pathways are simultaneously influenced by energy demand, mechanical loading, training status, and recovery. Changes in systemic antioxidant or inflammatory markers should therefore not be interpreted as direct evidence of pathway activation in exercising skeletal muscle [1,13].

Potential modulation may occur across several functional domains. Polyphenols and unsaturated fatty acids may influence the magnitude and resolution of NF-κB-dependent inflammatory responses, while EVOO phenolics and unsaturated lipids have been associated with endothelial homeostasis and nitric oxide bioavailability [5,7]. Food-derived metabolites may also affect the cellular environment in which AMPK–SIRT1–PGC-1α-dependent mitochondrial remodeling occurs. However, evidence that adherence to the complete MedDiet directly enhances these exercise-responsive pathways in human skeletal muscle remains limited. Thus, these mechanistic observations should be interpreted primarily as evidence of biological plausibility rather than as demonstration of pathway-specific effects of the MedDiet in humans. The most defensible interpretation is therefore that the dietary pattern may support the metabolic, vascular, and recovery conditions surrounding adaptation rather than directly reproduce or amplify every molecular signal generated by exercise.

Foundational exercise studies reinforce the need for an exposure-sensitive interpretation. The effects of reactive species on muscle function and adaptation depend on their source, concentration, localization, and duration [18,19,20]. Exercise-induced redox signaling operates within an integrated network involving energy stress, calcium flux, stress-responsive kinases, and mitochondrial regulation [21,22], while repeated endurance training increases mitochondrial content and oxidative enzyme capacity [23]. Repeated and recoverable perturbations can strengthen endogenous defenses, whereas excessive or unresolved stress may become maladaptive [24,25]. Conversely, studies of high-dose vitamins C and E indicate that concentrated antioxidant exposure can attenuate selected molecular responses to training, although effects on functional adaptation and performance are not uniform [26,27,28]. These findings do not argue against antioxidant-rich foods; they demonstrate that chemical form, dose, and exposure pattern influence the biological response.

The direction of nutritional modulation is also likely to be state-dependent. Individuals with low habitual intake of plant foods, insufficient nutrient status, high training loads, or inadequate recovery may respond differently from nutrient-replete and well-trained individuals. Similarly, an acute pre-exercise dose may produce a different effect from repeated exposure across meals, and a concentrated extract may not reproduce the pharmacokinetic profile of a whole food. The relationship between dietary bioactive exposure and adaptation should therefore be regarded as context-dependent and potentially nonlinear rather than summarized by a “more antioxidants, better adaptation” model [4,24,27,28].

Taken together, the available evidence is consistent with a model in which Mediterranean dietary bioactives may act primarily as exposure-dependent modulators rather than as simple systemic scavengers. Mechanistic plausibility is substantial, but the strength of evidence decreases when moving from chemical and cellular studies to tissue-specific pathway activation and functional adaptation in humans. Future trials should therefore characterize the dietary intervention, food matrix, circulating metabolites, training load, and sampling time within the same experimental design. The proposed sequence linking food composition, internal exposure, redox-sensitive signaling, and exercise-related outcomes is summarized in Figure 2 and provides the basis for the following discussion of bioavailability and interindividual variability.

The main Mediterranean dietary components, their predominant food sources, biologically relevant internal exposures, and proposed exercise-related mechanisms are summarized in Table 1.

**Table 1:** Major Mediterranean diet components, biologically relevant exposures, and proposed mechanisms potentially involved in exercise-related adaptation.

MedDiet Component/Exposure	Main Food Sources	Relevant Metabolites/Internal Exposure	Proposed Exercise-Related Mechanisms	Evidence/Interpretation	References
Polyphenols	EVOO, fruits, vegetables, legumes, whole grains	Phase II conjugates; microbial phenolic metabolites; phenolic acids; phenyl-γ-valerolactones; urolithins	May modulate redox-sensitive signaling, inflammatory regulation, endothelial function, and the cellular environment associated with mitochondrial remodeling	Strong mechanistic plausibility; direct evidence for pathway-specific effects of the complete MedDiet in exercising humans remains limited	[3,5,6,29,30,31,32,33,34,35,36,37,38]
Unsaturated fatty acids	EVOO, nuts, fish	Circulating fatty acids and lipid-derived mediators, including oxylipins, resolvins, and protectins	May influence membrane composition, inflammatory signaling, endothelial function, and vascular responses	Supported by mechanistic and systemic human evidence, but difficult to isolate from the whole-diet context	[7,8,9]
Fermentable dietary fiber	Legumes, whole grains, fruits, vegetables	Short-chain fatty acids, particularly acetate, propionate, and butyrate	May contribute to intestinal integrity, immune regulation, substrate metabolism, and cellular energy-sensing pathways	Biologically plausible; causal evidence linking SCFAs to exercise adaptation remains limited	[6,39,40,41,42,43]
Micronutrients	Fruits, vegetables, legumes, nuts, whole grains, fish	Vitamins, minerals, and related cofactor-dependent metabolic exposures	Support endogenous antioxidant defenses, energy metabolism, mitochondrial function, and general nutritional adequacy	Effects are context-dependent and may be most relevant when baseline nutritional status is suboptimal	[4,8,9]
Whole-food matrix/mixed MedDiet exposure	Combined Mediterranean dietary pattern	Repeated mixed exposure to host- and microbiota-derived metabolites	May integrate redox, metabolic, inflammatory, vascular, and microbial effects without reproducing the pharmacokinetic profile of isolated compounds	Most physiologically relevant exposure, but individual components and mechanisms cannot be attributed independently	[6,7,8,9]
Standardized extracts/concentrated bioactives	Concentrated food-derived preparations	Higher and more rapid circulating exposure, depending on formulation and dose	May affect selected redox-sensitive or inflammatory pathways, but may differ substantially from whole-food exposure	Useful for mechanistic studies; should not be considered biologically equivalent to the MedDiet or whole foods	[4,5,26,27,28,29,30]

Abbreviations: EVOO, extra-virgin olive oil; MedDiet, Mediterranean diet; SCFAs, short-chain fatty acids.

## 5. From Food Composition to Internal Exposure: Bioavailability, Food Matrix, and Interindividual Variability

The biological relevance of Mediterranean dietary polyphenols cannot be inferred from food composition tables or total antioxidant capacity alone. Bioavailability is highly compound-specific: glycosylation, esterification, molecular size, polymerization, and interactions with other food constituents influence release from the matrix, intestinal transport, and metabolic fate. Anthocyanins, flavan-3-ols, flavonols, phenolic acids, and secoiridoids differ substantially in absorption, phase II conjugation, biliary recycling, and microbial transformation. Many native polyphenols are poorly absorbed as intact molecules and, following uptake, undergo extensive glucuronidation, sulfation, and methylation in enterocytes and the liver. Plasma and tissues are therefore exposed predominantly to conjugated or lower-molecular-weight metabolites rather than to the parent aglycones commonly used in experimental studies [29,30,31,32,33,34]. Cell-based experiments employing unmetabolized compounds at concentrations substantially exceeding those achieved in humans may consequently overestimate direct antioxidant or signaling effects. Accordingly, mechanistic effects observed with native compounds in vitro should not be assumed to reflect the molecular effects of the circulating metabolites actually present in humans. The food matrix is an active determinant of exposure rather than an inert vehicle. Plant cell walls can limit or delay the release of phenolic compounds, whereas mastication, cooking, fermentation, and other processing procedures may increase extractability or promote oxidation and degradation. Lipids may facilitate the intestinal handling of relatively hydrophobic constituents, while proteins and dietary fiber can bind polyphenols, alter gastrointestinal transit, or redirect a larger fraction toward the colon. An olive-oil phenolic, apple flavanol, or anthocyanin consumed as part of a meal may therefore produce a substantially different pharmacokinetic profile from the same nominal dose administered as a purified compound. Standardized extracts provide a more reproducible but generally larger bolus exposure, whereas whole foods generate repeated exposures shaped by coexisting nutrients, food structure, digestion, and microbial metabolism [6,29,30]. Botanical and technological variability further complicates the translation from food composition to biological exposure. Cultivar, growing conditions, edible tissue, maturation, extraction, storage, and processing can modify both the concentration and profile of phenolic compounds. Studies of Annurca apples and their by-products have shown that reddening, ripening, and the anatomical part analyzed substantially influence phenolic composition and measured antioxidant activity [15,16]. EVOO provides another relevant example because cultivar, ripeness, extraction conditions, storage, meal composition, and the balance between free hydroxytyrosol and complex secoiridoid derivatives can alter the compounds available for absorption. Human pharmacokinetic evidence for hydroxytyrosol and analytical characterization of Italian EVOOs support treating both “apple polyphenols” and “olive-oil phenolics” as chemically heterogeneous exposures rather than uniform doses [35,36]. These analytical findings are essential for intervention standardization, but they do not independently establish systemic bioefficacy or exercise-related benefits.

A substantial proportion of unabsorbed polyphenols reaches the colon, where microbial deglycosylation, ring fission, reduction, and dehydroxylation generate smaller molecules, including phenolic acids, phenyl-γ-valerolactones, and urolithins. These compounds can subsequently be absorbed and further conjugated by the host. Some microbial metabolites persist longer in circulation than their dietary precursors and may contribute to vascular, inflammatory, metabolic, or redox-sensitive effects attributed to poorly absorbed parent compounds [37,38]. Their production, however, is not uniform. Urolithin formation from ellagitannins is the best-characterized example of distinct microbial metabotypes, but comparable interindividual differences affect the metabolism of several flavonoids and phenolic acids [44,45,46]. Individuals consuming the same food and nominal dose may therefore generate markedly different internal exposures.

Dose and timing must consequently be interpreted in relation to both pharmacokinetics and the exercise outcome under investigation. A concentrated dose administered shortly before exercise produces an exposure profile that is not equivalent to habitual intake distributed across meals. Small-intestinal metabolites may reach peak concentrations within hours, whereas microbiota-derived products generally appear later and may persist for longer periods. The relevant exposure window may therefore differ for acute vascular responses, substrate utilization, exercise-induced signaling, post-exercise inflammation, and recovery. Dose–response relationships may also be nonlinear because increasing the ingested dose can saturate absorption or conjugation, redirect more substrate toward the colon, and change the relative contribution of host- and microbiota-derived metabolites [29,37,38]. High-dose extracts should not be assumed to reproduce the biological effects of polyphenol-rich foods or a Mediterranean dietary pattern.

Interindividual variability reflects the combined influence of baseline microbiota, habitual diet, sex, age, genotype, medication use, intestinal transit, metabolic phenotype, and training status. These factors can modify compound release, absorption, enzymatic transformation, microbial metabolism, clearance, and tissue responsiveness. Consequently, an apparent non-response may result from inadequate internal exposure rather than absence of biological activity, whereas an average positive effect may be driven by a subgroup with a favorable metabolic or microbial phenotype. Food-composition resources such as Phenol-Explorer can improve the estimation and reporting of dietary polyphenol intake, but databases cannot replace direct characterization of the intervention batch or measurement of circulating and urinary metabolites [44,45,46,47,48].

Exercise-nutrition studies should therefore distinguish the administered dose from the internal bioactive exposure. At a minimum, interventions should report the food source, cultivar and processing conditions when relevant, complete polyphenol or bioactive profile, extraction method, food matrix, co-ingested macronutrients, dose, timing relative to exercise, treatment duration, adherence, background diet, and washout procedures. Whenever feasible, dietary assessment should be combined with batch analysis, targeted or untargeted metabolomics in plasma and urine, and repeated sampling capable of capturing both early absorption and delayed microbial transformation. Microbiome or stool-metabolite measurements may further help identify biotransformation phenotypes. This integrated approach may help distinguish inadequate exposure from biological non-response and provide a more rigorous basis for linking Mediterranean foods or bioactives to exercise adaptation. The sequence connecting food composition, matrix-dependent release, host and microbial metabolism, internal exposure, and potential exercise-related outcomes is summarized in Figure 2.

## 6. Human Evidence Across Mediterranean Dietary Patterns and Related Food-Based Interventions

Human evidence directly testing whether the MedDiet modifies exercise performance, recovery, or training adaptation remains limited compared with the mechanistic literature. Recent systematic and scoping reviews have identified relatively few controlled interventions, with substantial heterogeneity in dietary definition, participant phenotype, training status, comparator diet, intervention duration, and outcome selection [8,9]. Interpretation is further complicated by the inclusion of three non-equivalent exposure categories: trials comparing a MedDiet with a non-Mediterranean dietary pattern; multifactorial interventions in which diet, physical activity, energy intake, and body mass change concurrently; and studies of individual foods, extracts, or isolated bioactives. Only the first category directly evaluates the dietary pattern, whereas the others provide supportive but indirect evidence regarding diet–exercise interactions.

One of the clearest short-term performance signals was reported in a randomized-sequence crossover trial involving 11 recreationally active adults. After four days of a MedDiet or a Western diet, participants completed endurance and anaerobic performance tests. MedDiet improved 5-km treadmill performance by approximately 6%, whereas no significant advantages were observed for Wingate performance, vertical jump, or handgrip strength [49]. The within-participant design reduced between-subject variability, but the small sample and brief dietary exposure limit the strength and generalizability of the finding. Moreover, several nutritional characteristics—including carbohydrate availability, nitrate intake, micronutrient adequacy, and acid–base load—may have differed simultaneously. The study therefore provides evidence of an acute whole-diet effect on a specific endurance endpoint, but does not establish enhanced VO_2_max, mitochondrial remodeling, or long-term training adaptation.

Evidence for strength-related outcomes is less persuasive. Perna et al. investigated 13 non-professional strength athletes using a randomized crossover design comprising two eight-week Mediterranean dietary phases that provided approximately 55–60% or 40–45% of energy from carbohydrate [50]. Most measures of strength, physical performance, body composition, and circulating biomarkers did not differ significantly between conditions; elbow-flexor maximal voluntary contraction represented the principal exception. Because both interventions followed Mediterranean-style dietary principles, the study evaluated carbohydrate distribution within the MedDiet rather than the superiority of the MedDiet over a non-Mediterranean comparator. The small sample and number of outcomes also require caution when interpreting an isolated positive result.

Among the studies identified in this review, the most direct test of MedDiet-induced training adaptation is a 12-week randomized trial conducted in 60 competitive endurance athletes, of whom 55 completed the intervention. Participants followed a standardized endurance-training program and were assigned to either a MedDiet or a control diet. Improvement in VO_2_max was greater in the MedDiet group, with an adjusted between-group difference of 1.1 mL/kg/min. The intervention also increased plasma propionate and butyrate, and changes in these metabolites were associated with improvements in VO_2_max [10]. This study is notable because it connects a whole dietary pattern, controlled training, microbial characteristics, circulating metabolites, and a functional endpoint. However, associations and statistical mediation do not demonstrate that short-chain fatty acids caused performance improvement. Differences in carbohydrate intake, energy availability, micronutrient exposure, or other dietary components may also have contributed, and replication with detailed assessment of training load and internal metabolite exposure is required. Thus, the study provides important human support for the proposed framework but does not establish a causal microbiota–metabolite–performance pathway.

Longer interventions have primarily involved clinical rather than athletic populations and should therefore be interpreted as evidence of systemic effects rather than sport-specific efficacy. In a 12-month randomized study of 124 participants with obesity, a MedDiet combined with physical activity was compared with a standard hypolipidemic diet combined with physical activity; 84 participants completed the intervention. Serum total antioxidant capacity initially increased in both groups but was better maintained in the MedDiet group, resulting in a significant time-by-diet interaction [51]. Although this finding supports a sustained effect on a circulating antioxidant index, total antioxidant capacity neither identifies a specific redox pathway nor demonstrates improved training adaptation. Similarly, a six-month PREDIMED-Plus substudy involving 391 older adults with metabolic syndrome found that an energy-restricted MedDiet combined with physical activity improved aspects of HDL triglyceride metabolism compared with a non-energy-restricted MedDiet without an exercise program [52]. Because dietary energy restriction, physical activity, and body mass changed concurrently—and weight loss mediated much of the observed effect—the independent contribution of MedDiet–exercise crosstalk cannot be isolated.

Evidence from individual foods or preparations provides useful proof of concept but should not be conflated with evidence for the complete dietary pattern. Dietary nitrate has been shown to reduce the oxygen cost of submaximal exercise and improve exercise tolerance or selected performance outcomes, although responsiveness may be smaller and less consistent in highly trained athletes [53,54,55]. Tart cherry and blueberry interventions have produced improvements in selected indices of soreness, inflammation, oxidative stress, or functional recovery after demanding exercise [56,57,58,59]. Reviews and meta-analyses similarly suggest that some polyphenol-rich foods or preparations can modestly support performance or recovery from exercise-induced muscle damage, with effects varying according to dose, food source, damage model, training status, and outcome timing [56,59,60,61,62]. These findings generate hypotheses regarding specific components or metabolites, but they do not demonstrate that habitual adherence to the MedDiet produces the same exposure or effect. Concentrated juices, extracts, and supplements may deliver doses and pharmacokinetic profiles that differ substantially from those obtained through whole foods consumed as part of a mixed dietary pattern.

Evidence for post-exercise recovery remains particularly limited at the whole-diet level. MedDiet interventions have rarely incorporated repeated assessments of muscle soreness, force restoration, creatine kinase, inflammatory resolution, sleep, perceived recovery, or readiness to train [8,9,60]. This represents an important gap because an isolated reduction in an oxidation or inflammatory marker cannot establish faster functional recovery and may not necessarily indicate a more favorable training response. Trial duration and participant phenotype are also critical. Studies lasting several days primarily examine acute substrate availability, vascular responses, gastrointestinal tolerance, or immediate performance, whereas interventions lasting weeks or months are required to assess VO_2_max, lactate thresholds, strength development, body composition, mitochondrial adaptation, and training continuity. Responses may also differ between untrained individuals, recreationally active adults, and highly trained athletes operating close to a performance ceiling.

Observational studies can help describe dietary practices and identify candidate associations but cannot establish ergogenic efficacy. Mediterranean-diet adherence has been examined in competitive athletes, including participants in the XXX Summer Universiade, but observed relationships remain vulnerable to residual confounding by sport discipline, sex, energy expenditure, socioeconomic conditions, and health-conscious behavior [63]. Adherence scores are also imperfect proxies for bioactive exposure because individuals with similar scores may consume different foods, cultivars, quantities, processing methods, and total energy. Large Mediterranean-diet trials provide strong contextual evidence for cardiovascular safety and systemic relevance, but they were not designed to isolate exercise adaptation or polyphenol action in athletic populations [64,65].

Biomarker interpretation requires comparable methodological discipline. Total antioxidant capacity is influenced by urate and other circulating constituents and cannot independently establish reduced oxidative damage. More informative assessment should combine markers addressing different biological compartments, such as F_2_-isoprostanes or other validated lipid-oxidation products, protein carbonyls, oxidized nucleic-acid products, glutathione redox status, and inflammatory mediators. When feasible, these measurements should be complemented by skeletal-muscle signaling, mitochondrial function, or endothelial assessments [66,67]. Sampling schedules must reflect biomarker- and exercise-specific kinetics because peak responses may occur immediately after exercise or several hours to days later. Functional outcomes, including performance, restoration of force, illness burden, and training continuity, should remain primary, with biomarkers used to explain rather than replace them. This distinction is important when separating mechanistic plausibility from demonstrated functional benefit in humans.

Overall, current human evidence is consistent with possible modest benefits for selected endurance, VO_2_max, cardiometabolic, and antioxidant outcomes, but it does not support a uniform ergogenic effect of the MedDiet. Findings for maximal strength are predominantly neutral, and direct evidence for accelerated recovery remains insufficient. Future trials should be adequately powered and preregistered, recruit clearly characterized athletic populations, standardize or objectively monitor training load, and compare a well-defined MedDiet with a credible isoenergetic and carbohydrate-matched control. Dietary adherence should be supported by intervention-batch characterization and measurement of circulating food- and microbiota-derived metabolites. Repeated assessments of performance, recovery, redox and inflammatory responses, and metabolite exposure would help determine whether observed effects reflect the dietary pattern itself, improved nutritional adequacy, changes in energy balance or body mass, or potential modulation of exercise-responsive pathways. Representative human interventions and their principal interpretative limitations are summarized in Table 2.

## 7. Microbial Biotransformation as a Link Between the Mediterranean Diet and Exercise Adaptation

The gut microbiota may contribute to linking Mediterranean dietary exposure with exercise adaptation through a bidirectional gut–muscle axis. Exercise can modify the intestinal environment, microbial composition, and metabolic activity, whereas microbial metabolites and immune signals may influence host metabolism, inflammation, intestinal integrity, and skeletal-muscle function [39,68,69]. The relevant mechanistic unit is therefore not a single bacterial taxon but the sequence linking dietary substrates, microbial functions, circulating metabolites, and host responses.

Most evidence from athletes remains associative. Elite athletes frequently show greater microbial diversity and functional capacity than sedentary individuals, but these differences are confounded by training load, diet, energy intake, body composition, travel, and medication use [70,71]. Controlled studies indicate that exercise can modify microbial composition and short-chain fatty acid (SCFA)-related pathways, with responses differing according to metabolic phenotype and partially reversing after detraining [72,73]. These findings support a dynamic ecosystem shaped jointly by exercise and diet rather than a universal “athletic microbiome.”

The MedDiet is relevant because it provides fermentable dietary fiber and polyphenols that are incompletely absorbed in the small intestine. In the colon, fermentable fibers are metabolized by the microbiota to SCFAs, whereas polyphenols undergo microbial biotransformation into smaller phenolic metabolites with potential systemic activity [6,40,41,42]. This substrate-to-metabolite perspective is more informative than taxonomic profiling alone because different microbial communities may generate similar metabolites, whereas modest compositional changes may produce substantial functional effects.

Acetate, propionate, and butyrate may influence epithelial integrity, immune regulation, substrate metabolism, and pathways related to cellular energy sensing and mitochondrial function [39,43]. Microbial transformation of polyphenols also produces phenolic acids and metabotype-dependent metabolites that may contribute to vascular, inflammatory, and redox-sensitive effects [41,42]. However, fecal SCFA concentrations reflect the net balance among production, utilization, and absorption, while plasma concentrations represent only the circulating fraction. Neither measurement alone demonstrates delivery to skeletal muscle or activation of a specific signaling pathway.

The most direct human evidence is provided by the 12-week trial by Lu et al. [10]. In competitive endurance athletes, the MedDiet increased microbial alpha diversity, selected SCFA-producing taxa, and plasma propionate and butyrate, while producing a greater improvement in VO_2_max than the control diet. Propionate statistically mediated part of the dietary effect. Nevertheless, mediation analysis cannot establish causality, and the simultaneous changes in fiber, polyphenols, fatty acids, micronutrients, and overall diet quality prevent attribution to a single metabolite. Moreover, 16S rRNA sequencing provides limited information about microbial genes, strain-level variation, and metabolic flux. Thus, these findings support an association between dietary modulation, microbial changes, circulating metabolites, and training-related outcomes, but do not establish a causal microbial pathway.

Interindividual variability further limits generalization. Baseline microbiota, habitual diet, sex, medication and antibiotic exposure, intestinal transit, metabolic health, and training status can influence both metabolite production and responsiveness to dietary change [42,43,68]. The same dietary precursor may therefore generate different circulating metabolites in different individuals. Training load is also relevant because prolonged strenuous exercise, inadequate energy availability, heat stress, and insufficient recovery may disrupt gastrointestinal homeostasis and modify tolerance to fiber-rich foods [68]. Microbiota-directed strategies must consequently be considered within the athlete’s overall dietary, training, and recovery context.

Future trials should combine controlled MedDiet interventions with objective monitoring of diet and training, repeated stool and blood sampling, metagenomic or metatranscriptomic analysis, and targeted metabolomics. Measurements of intestinal permeability, inflammation, skeletal-muscle signaling, mitochondrial function, and performance would help distinguish microbial associations from functional mediation. Baseline microbial and metabolite profiles may help identify responder phenotypes and support more precise dietary interventions.

Overall, microbiota-derived metabolites represent a plausible but incompletely established component of MedDiet–exercise interactions. Current evidence supports microbiota as a potential mediator of dietary exposure, but not as an independent or proven determinant of performance. Its contribution should therefore be interpreted as one component of a broader network that also includes metabolic, redox, inflammatory, and vascular effects of the dietary pattern.

## 8. Food-Based Redox Modulation Versus High-Dose Antioxidant Supplementation

The nutritional effect of an antioxidant cannot be predicted from its chemical classification alone. Exercise-induced reactive oxygen and nitrogen species contribute to adaptation when their production is transient and controlled, whereas excessive or unresolved oxidative stress may impair recovery and cellular function. Nutritional interventions should therefore be evaluated according to their ability to modulate redox signaling without suppressing the signals required for endogenous defense and mitochondrial remodeling [24,74].

Human trials of vitamins C and E provide the clearest evidence that high-dose antioxidant supplementation may modify exercise responses. Ristow et al. [75] reported that four weeks of vitamin C and E supplementation attenuated improvements in insulin sensitivity and the induction of PGC-1α and endogenous antioxidant enzymes. These findings support a role for exercise-generated oxidants in metabolic adaptation. However, the study tested pharmacological doses of isolated vitamins over a short period and should not be extrapolated to antioxidant-rich foods.

Paulsen et al. [76] similarly found that vitamin C and E supplementation attenuated increases in selected mitochondrial proteins during 11 weeks of endurance training, without impairing improvements in VO_2_max or running performance. Other trials have also produced divergent molecular and functional findings [26,27,28]. A supplement may therefore alter a signaling pathway without causing a measurable performance loss during the study period. Conversely, an unchanged systemic biomarker does not exclude tissue-specific effects.

Differences among studies probably reflect variation in dose, formulation, timing, baseline nutritional status, training modality, intervention duration, and outcome selection [27,74,77]. Correcting a deficiency is biologically different from administering high doses to a well-nourished athlete. Similarly, supplementation immediately before or after exercise may affect acute redox signaling differently from nutrients consumed across meals. Evidence does not support a universal detrimental effect, but it does question the routine use of chronic high-dose antioxidant supplementation when no deficiency or specific clinical indication is present.

Isolated polyphenols represent a more heterogeneous exposure. Their biological actions may include modulation of kinases, transcription factors, inflammatory pathways, endothelial signaling, and endogenous antioxidant defenses rather than direct radical scavenging alone [5,29,78]. Nevertheless, purification and concentration can transform a food constituent into a supplement-like exposure. Circulating forms are predominantly conjugated or microbiota-derived metabolites, rather than the parent compounds frequently used in cell studies [29,30,35,37,38]. The effects of an extract or purified polyphenol therefore cannot be inferred solely from chemical antioxidant capacity or experiments employing physiologically unattainable concentrations.

Whole foods provide a different exposure pattern. Fruits, vegetables, nuts, legumes, whole grains, and extra-virgin olive oil deliver moderate amounts of multiple bioactives together with fiber, unsaturated lipids, minerals, and other nutrients. The food matrix influences compound release, absorption, metabolism, and microbial transformation, producing lower and more sustained exposures than concentrated supplements [6,41]. These foods may support nutritional adequacy and may help modulate excessive oxidative or inflammatory responses without creating the rapid antioxidant exposure associated with high-dose preparations. This possibility, however, should be tested rather than assumed.

The same distinction applies to the Med Diet. Its potential effects derive from the combined dietary pattern and cannot be attributed exclusively to polyphenols or antioxidant vitamins. Current human studies suggest possible benefits for selected performance and antioxidant outcomes, but controlled evidence remains limited and heterogeneous [2,8,9]. Improvements may also reflect changes in carbohydrate availability, fatty-acid composition, micronutrient intake, energy balance, or overall diet quality. The available data therefore support compatibility with exercise adaptation, not a consistently demonstrated antioxidant-mediated ergogenic effect.

A food-first approach is consequently more defensible than routine high-dose supplementation. This does not exclude targeted supplementation to correct deficiency or address a defined nutritional requirement, but dose, timing, product composition, safety, and training context should be considered [79]. Concentrated extracts should not automatically be classified as food-equivalent merely because their active compounds originate from plants.

Future trials should directly compare Mediterranean dietary patterns, whole foods, standardized extracts, and isolated antioxidants under the same exercise protocol. Measurement of circulating metabolites, redox-sensitive signaling, recovery, and performance would clarify whether differences reflect dose, matrix, pharmacokinetics, or compound-specific activity. The central distinction is therefore not between “antioxidants” and “oxidants,” but between physiologically relevant food-based exposure and concentrated supplementation that may alter exercise-responsive signaling.

From a practical perspective, Mediterranean dietary exposures are more appropriately integrated through habitual meals containing fruits, vegetables, legumes, whole grains, nuts, EVOO, and fish rather than through attempts to reproduce the dietary pattern with concentrated antioxidant supplements. The timing and relevance of specific foods or bioactive-rich preparations may nevertheless depend on training modality, exercise intensity, gastrointestinal tolerance, energy availability, and the intended outcome (e.g., acute performance, recovery, or long-term adaptation).

## 9. Toward Precision Redox Nutrition: A Methodological Roadmap for Diet–Exercise Studies

The next advance in this field will not come from classifying foods and supplements as simply antioxidant or pro-oxidant. In this review, precision redox nutrition refers to matching a defined dietary exposure with the individual’s bioavailability, microbial biotransformation, baseline redox phenotype, and exercise stimulus. It does not imply that every athlete requires a unique diet; rather, it identifies measurable sources of variability that should be incorporated into study design. The relevant experimental sequence extends from the ingested food, matrix, and dose to circulating metabolites, redox-sensitive responses, potential functional adaptation, and performance.

Dietary exposure must first be characterized accurately. MedDiet interventions should report the foods provided or prescribed, processing and cultivar when relevant, bioactive composition, background diet, adherence, and supplement use. Adherence scores and food-frequency questionnaires describe dietary patterns but cannot determine internal exposure to specific metabolites. Where feasible, plasma or urinary metabolomics should therefore be used to confirm exposure and identify conjugated and microbiota-derived compounds. Repeated sampling is preferable to a single fasting measurement because metabolite concentrations vary with meal timing, absorption, enterohepatic circulation, and microbial transformation [80]. Failure to detect a parent compound does not demonstrate absence of exposure, and identical intakes cannot be assumed to produce identical internal doses.

Redox assessment should likewise be dynamic rather than based on a single antioxidant marker. Total antioxidant capacity and isolated measurements of superoxide dismutase, catalase, or glutathione provide incomplete and assay-dependent information. Studies should combine complementary measures of oxidative modifications, antioxidant defenses, and redox couples, such as F_2_-isoprostanes, protein carbonyls, reduced and oxidized glutathione, and selected enzymatic activities [66,67]. Specimen type, collection, storage, analytical validation, and normalization should be reported. Sampling must also follow biomarker-specific kinetics because responses may occur immediately after exercise or emerge over the subsequent 24–72 h. Functional outcomes should remain primary, with biomarkers used to investigate mechanism rather than substitute for performance or recovery.

Training modality, external workload, internal physiological response, fitness level, recovery interval, energy availability, hydration status, environmental heat exposure, and recent training history can influence redox signaling and adaptation [19,22,81]. Acute studies are useful for defining the temporal relationship between metabolite exposure and molecular responses, whereas longitudinal trials are required to determine whether repeated exposure modifies training adaptation. Molecular endpoints—including Nrf2-related targets, AMPK–SIRT1–PGC-1α signaling, mitochondrial proteins, and inflammatory mediators—should therefore be examined alongside reliable measures of VO_2_max, strength, substrate utilization, fatigue resistance, or recovery. This approach would also help establish whether the relevance of a dietary exposure depends on its timing relative to exercise and on the specific training context.

Microbial assessment should focus on function rather than taxonomy alone. Shotgun metagenomics, microbial pathway analysis, fecal metabolomics, and circulating metabolite measurements can help determine whether changes in microbial composition are associated with changes in host exposure. Individuals may differ in their ability to generate urolithins, phenyl-γ-valerolactones, and other microbial products despite comparable dietary adherence. These metabotypes may represent useful stratification variables, but their relationship with intervention response must be prospectively tested and supported by direct metabolite measurements [42,82].

An informative trial design would combine a standardized run-in period, controlled or well-documented dietary exposure, monitored training, and repeated biological sampling. Factorial designs could compare a MedDiet with a whole food, standardized extract, or isolated bioactive under the same training program, thereby separating dietary-pattern, food-matrix, and compound-specific effects. Crossover designs may be appropriate for acute outcomes, whereas parallel-group trials are preferable when diet and exercise are expected to produce durable metabolic or microbial adaptations.

A minimum dataset should include participant characteristics, habitual diet, training status, medication and supplement use, intervention composition, dose, timing, adherence, adverse events, and objective measures of training load. Trials should define a reliable primary functional outcome and select mechanistic endpoints according to a prespecified causal model. Repeated measures or mixed-effects models can describe individual trajectories, while correction for multiple comparisons is essential when several biomarkers or omics features are examined. Candidate moderators, including sex, baseline diet, training status, redox phenotype, and microbial metabotype, should be measured before allocation and tested through prespecified interactions rather than post hoc subgroup analyses.

Multi-omics and machine-learning approaches may help identify response signatures, but prediction must be distinguished from biological explanation. Adequate sample size, independent validation, transparent feature selection, and appropriate handling of missing data are required to limit overfitting. Personalized nutrition trials have demonstrated feasibility and selected cardiometabolic benefits, but they do not yet establish that algorithm-based personalization improves exercise adaptation or redox regulation [83].

The proposed roadmap prioritizes causal resolution rather than technological complexity. A study need not include every omics platform, but it should connect a well-defined dietary exposure with an internal dose, a temporally appropriate biological response, and a meaningful functional outcome. This framework could help clarify which Mediterranean foods or bioactives may provide measurable benefits, for whom, at what dose and timing, and under which training conditions.

## 10. Conclusions

The biological effects of dietary bioactives in the context of exercise cannot be predicted from antioxidant capacity or food composition alone. This review supports an exposure-aware interpretation in which the effects of Mediterranean dietary bioactives depend on food matrix, chemical form, dose, timing, intestinal absorption, host metabolism, and microbial biotransformation. Consequently, the administered compound and the biologically relevant exposure are not equivalent: tissues are exposed predominantly to circulating conjugated and microbiota-derived metabolites rather than to the native compounds commonly investigated in experimental systems.

Within this framework, MedDiet should be considered a complex and repeated food-based exposure rather than a source of isolated antioxidants. Polyphenols, unsaturated lipids, fiber, micronutrients, and their host- and microbiota-derived metabolites may modulate the redox, energy-dependent, inflammatory, and vascular environment in which exercise adaptation occurs. Importantly, exercise-generated RONS are integral components of adaptive signaling; therefore, the nutritional objective should not be their indiscriminate suppression but the maintenance of an appropriate redox–metabolic response. Whole foods, standardized extracts, and high-dose supplements should consequently not be regarded as biologically interchangeable. High-dose antioxidant supplementation may attenuate selected molecular responses, whereas evidence for a comparable effect of antioxidant-rich foods is lacking.

Current human evidence remains insufficient to establish a consistent ergogenic effect of the MedDiet. Available studies suggest possible benefits for selected endurance-related, metabolic, vascular, and recovery-related outcomes, although evidence for accelerated recovery remains limited. Findings are heterogeneous, and direct evidence linking specific circulating metabolites to molecular and functional training adaptations remains limited.

Future research should therefore move beyond the simple classification of foods and nutraceuticals as “antioxidant” and instead connect food composition → internal exposure → exercise-responsive signaling → functional outcome. Well-characterized interventions, direct measurement of circulating metabolites, repeated biological sampling, standardized training loads, and meaningful functional endpoints will be essential to distinguish inadequate exposure from true biological non-response and to clarify which Mediterranean foods or bioactives may provide measurable benefits, at what dose and timing, in which individuals, and under which training conditions. Such an approach provides a mechanistic and methodological basis for advancing precision redox nutrition in exercise.

## Figures and Tables

**Figure 1:**
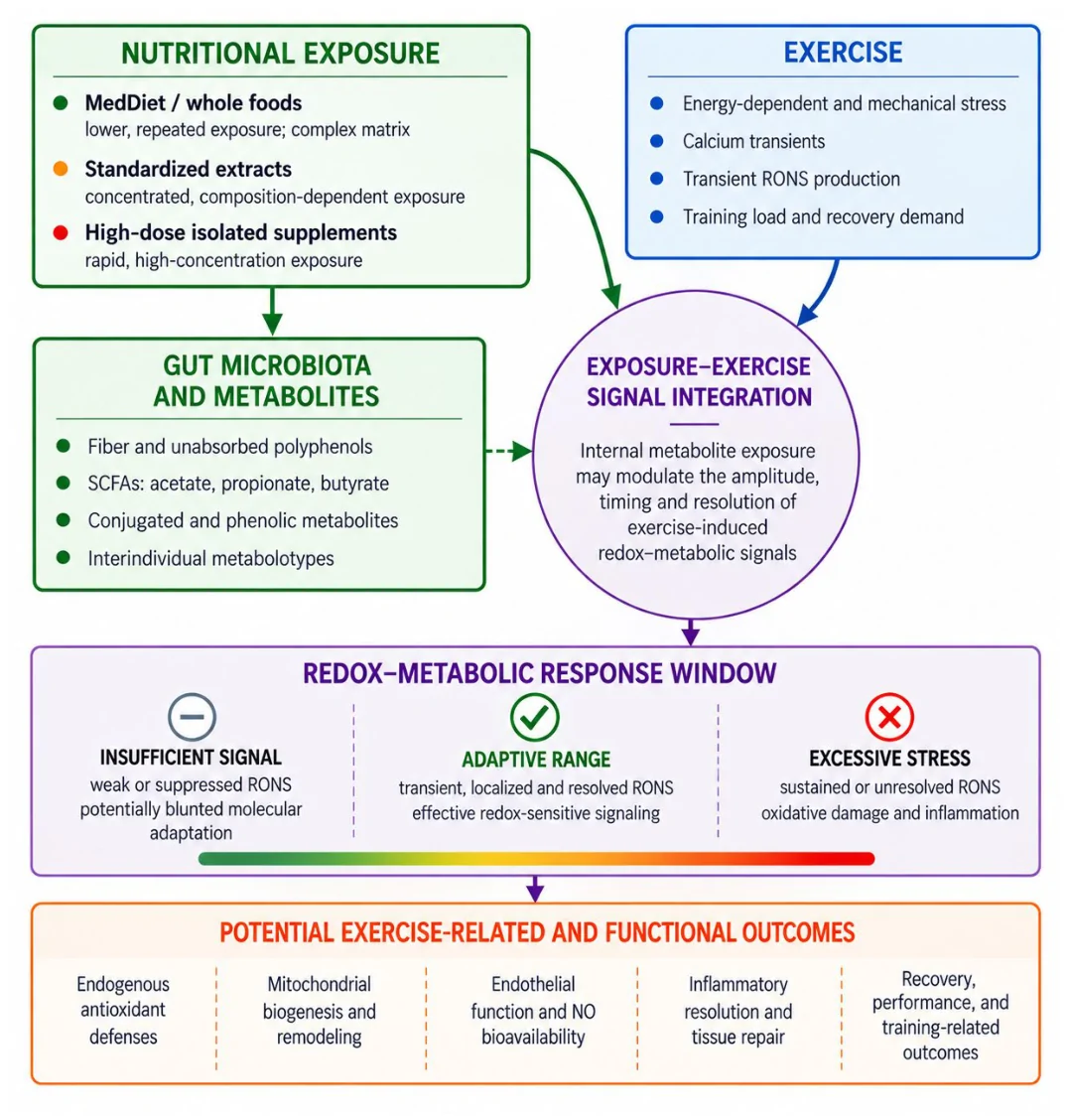
Nutritional exposure, redox signaling, and exercise adaptation. Different nutritional exposures, from Mediterranean diet/whole foods to standardized extracts and high-dose supplements, may modulate exercise-induced redox and metabolic signaling directly or through gut microbiota-derived metabolites. An optimal, transient RONS response supports adaptation, whereas insufficient signaling may blunt adaptation and excessive stress may promote oxidative damage and inflammation. The resulting response may contribute to mitochondrial, vascular, inflammatory, recovery, and performance-related adaptations. Arrows represent conceptual or proposed relationships and should not be interpreted as evidence of causality unless supported by controlled human studies.

**Figure 2:**
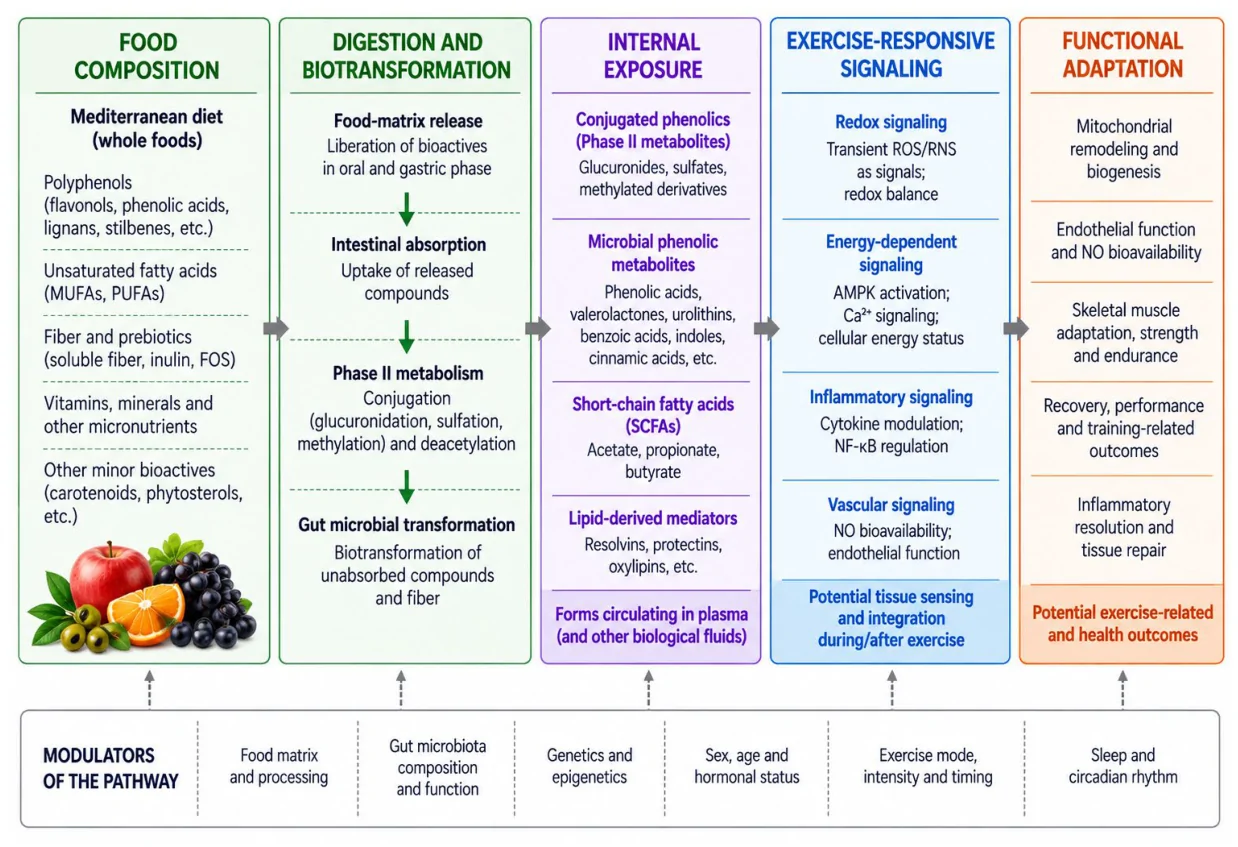
From food composition to potential exercise-related outcomes: metabolic and signaling pathway. Mediterranean diet components are released from the food matrix, absorbed, transformed by host metabolism and gut microbiota, and converted into circulating metabolites. These internal exposures may interact with exercise-responsive redox, energy-dependent, inflammatory, and vascular signaling and may thereby contribute to mitochondrial remodeling, endothelial function, muscle adaptation, recovery, and performance-related outcomes.

**Table 2:** Representative human interventions examining Mediterranean dietary patterns and exercise-related outcomes.

Study and Design	Intervention	Main Findings	Interpretation
Baker et al. [49]; 11 active adults; crossover	4-day MedDiet vs. Western diet	~6% faster 5-km performance; no significant benefit for other performance outcomes	Promising acute finding, but limited by the very small sample and short intervention
Perna et al. [50]; 13 strength athletes; crossover	Two 8-week MedDiets differing in carbohydrate content	Most outcomes unchanged; isolated improvement in elbow-flexor strength	Does not compare the MedDiet with a non-Mediterranean dietary pattern
Lu et al. [10]; 60 endurance athletes; 12-week randomized trial	MedDiet plus training vs. control diet plus training	Greater increase in VO_2_max; higher plasma propionate and butyrate	Most direct evidence for a MedDiet–training interaction; causal mediation by SCFAs remains unproven
Ortner Hadžiabdić et al. [51]; 124 adults with obesity; 12-month randomized trial	MedDiet plus physical activity vs. hypolipidemic diet plus physical activity	Total antioxidant capacity better maintained	Demonstrates a systemic biomarker effect, but not exercise-specific training adaptation
Sanllorente et al. [52]; 391 adults with metabolic syndrome; 6-month substudy	Energy-restricted MedDiet plus physical activity vs. non-energy-restricted MedDiet without an exercise program	Improved aspects of HDL triglyceride metabolism	Multicomponent intervention; effects cannot be attributed specifically to MedDiet–exercise crosstalk

Abbreviations: HDL, high-density lipoprotein; MedDiet, Mediterranean diet; SCFAs, short-chain fatty acids; VO_2_max, maximal oxygen uptake.

## Data Availability

No new data were created or analyzed in this study. Data sharing is not applicable to this article.

## Supplementary Materials

The following supporting information can be downloaded at: https://www.mdpi.com/article/10.3390/biotech15040088/s1, Table S1: Comparison of the Present Review with Recent Reviews on Mediterranean Diet, Exercise Performance, Dietary Bioactives, and Gut Microbiota.

## Abbreviations

AMP	adenosine monophosphate
AMPK	AMP-activated protein kinase
ADP	adenosine diphosphate
ATP	adenosine triphosphate
EVOO	extra-virgin olive oil
HDL	high-density lipoprotein
Keap1	Kelch-like ECH-associated protein 1
MedDiet	Mediterranean diet
NAD^+^	nicotinamide adenine dinucleotide oxidized form
NADPH	reduced nicotinamide adenine dinucleotide phosphate
NF-κB	nuclear factor kappa B
Nrf2	nuclear factor erythroid 2-related factor 2
PGC-1α	peroxisome proliferator-activated receptor-γ coactivator 1α
RONS	reactive oxygen and nitrogen species
SCFAs	short-chain fatty acids
SIRT1	sirtuin 1
VO_2_max	maximal oxygen uptake
