# Supplementary material for: Beyond Antioxidant Scavenging: Mediterranean Diet, Redox–Metabolic Integration, and Exercise Adaptation
Source: BioTech (Basel). 2026 Oct 6;15(4):88. doi: 10.3390/biotech15040088 (PMC13702537; doi:10.3390/biotech15040088)
Supplement: Supplementary file 1 [file biotech-15-00088-s001.zip › biotech-4583942-supplementary.pdf]

BioTech

Supplementary Materials: Beyond Antioxidant Scavenging: Mediterranean Diet, Redox–Metabolic Integration, and Exercise Adaptation

Stefania D'Angelo

Table S1. Comparison of the Present Review with Recent Reviews on Mediterranean Diet, Exercise Performance, Dietary Bioactives, and Gut Microbiota.

Review	Review Type and Main Focus	Evidence Base/Principal Scope	Aspect Less Extensively Addressed	Distinctive Contribution of the Present Review
Bianchi et al., 2024	Systematic review of Mediterranean diet interventions in relation to athletic performance, muscle strength, body composition, and antioxidant markers	Intervention trials involving athletes and non-professional athletes	Limited integration of food matrix, bioavailability, microbial biotransformation, and circulating metabolite exposure	Integrates dietary exposure characteristics with internal exposure, redox–metabolic signaling, and exercise-related functional outcomes
Mantzioris et al., 2024	Scoping review examining the relationship between Mediterranean dietary pattern adherence and exercise or sport performance	Broad mapping of observational and intervention evidence related to MedDiet adherence, sport performance, and athlete health	Limited emphasis on pharmacokinetics, internal exposure, and mechanistic integration of host and microbial metabolism	Links the whole dietary pattern with food matrix, host and microbial biotransformation, circulating metabolites, and exercise-responsive signaling
Fiorini et al., 2025	Systematic review and meta-analysis evaluating Mediterranean diet and athletic performance in elite and competitive athletes	Studies of competitive/elite adult athletes, with quantitative synthesis of selected performance outcomes	Primary emphasis on performance outcomes rather than exposure-dependent molecular and metabolic mechanisms	Distinguishes mechanistic plausibility from demonstrated human efficacy and integrates performance evidence within an exposure-aware framework
Gaskell et al., 2026	Systematic review of nutritional, dietary, and exercise interventions targeting gut microbiota and subsequent exercise performance	Intervention studies examining microbiota-related responses to diet, supplements, and exercise	Not specifically focused on the Mediterranean dietary pattern or on the complete food-to-metabolite exposure pathway	Incorporates microbiota-derived metabolites as one component of a broader MedDiet–exercise framework integrating dietary source, internal exposure, redox–metabolic signaling, and potential functional outcomes
Present review	Structured narrative review integrating Mediterranean dietary patterns, dietary bioactives, bioavailability, gut microbiota, redox–	Mechanistic, preclinical, observational, and controlled human evidence, with separate consideration of whole dietary patterns, foods,	—	Proposes an exposure-aware framework connecting ingested dietary composition and food matrix with host and microbial biotransformation, circulating metabolites,

	metabolic signaling, and exercise adaptation	standardized extracts, and isolated supplements		exercise-responsive signaling, and potential functional outcomes, while explicitly distinguishing mechanistic plausibility from demonstrated human efficacy

Abbreviations: MedDiet, Mediterranean diet.

References

1. Bianchi, E.; Erbasan, H.; Riso, P.; Perna, S. Impact of the Mediterranean Diet on Athletic Performance, Muscle Strength, Body Composition, and Antioxidant Markers in Both Athletes and Non-Professional Athletes: A Systematic Review of Intervention Trials. *Nutrients* **2024**, *16*, 3454, https://doi.org/10.3390/nu16203454.
2. Mantzioris, E.; Villani, A.; Forsyth, A. The Relationship Between the Mediterranean Dietary Pattern and Exercise and Sport Performance—A Scoping Review. *Nutrients* **2024**, *16*, 4259, https://doi.org/10.3390/nu16244259.
3. Fiorini, S.; Guglielmetti, M.; Neri, L.C.L.; Correale, L.; Tagliabue, A.; Ferraris, C. *Mediterranean Diet and Athletic Performance in Elite and Competitive Athletes: A Systematic Review and Meta-Analysis.* Nutrition, Metabolism and Cardiovascular Diseases 2025, 35, 104165. https://doi.org/10.1016/j.numecd.2025.104165. (PubMed)
4. Gaskell, S.K.; Martinez, I.; Costa, R.J.S. *Gut Microbiota and Exercise: A Systematic Review of Interventions and Evidence Limitations.* International Journal of Sports Medicine 2026, 47, 255–286. https://doi.org/10.1055/a-2705-2210. (PubMed)
